# A renewable carbon material derived from native European deciduous trees serves as a sustainable electroactive substance for multifunctional energy storage systems†

**DOI:** 10.1039/d5na00018a

**Published:** 2025-04-14

**Authors:** Surjit Sahoo, Thiba Nagaraja, Monika Michalska, Suprem R. Das

**Affiliations:** a Department of Industrial and Manufacturing Systems Engineering, Kansas State University Manhattan KS 66506 USA srdas@ksu.edu surjit488@gmail.com; b Mechanical Engineering Department, Indian Institute of Technology Jammu 181221 Jammu & Kashmir India; c Department of Electrical and Computer Engineering, Kansas State University Manhattan KS 66506 USA; d Department of Chemistry and Physico-Chemical Processes, VSB-Technical University of Ostrava 17. listopadu 2172/15 70800 Ostrava-Poruba Czech Republic

## Abstract

Carbon derived from biomass, characterized by its abundant porosity and adaptable physical and chemical traits, has emerged as a promising choice for electrode materials in electrochemical energy storage devices like supercapacitors and lithium–sulfur (Li–S) batteries, marking a rapidly advancing field. Herein, we report the creation of a fresh biomass-derived activated carbon produced *via* a pyrolysis technique using a blend of indigenous European deciduous trees, including Birch, Fagaceae, and Carpinus betulus (commonly referred to as European hornbeam). The biomass-derived activated carbon underwent various material characterizations to scrutinize its structural, morphological, and elemental compositions. Utilizing this biomass-derived activated carbon as the electrode material across different supercapacitor configurations (such as coin cells and printable miniaturized devices) and as sulfur hosts in Li–S batteries paves the way for expanded applications in biomass energy utilization. The supercapacitor devices were successfully fabricated and shown to be operated efficiently within an operational potential range of 2.5 V (0.0–2.5 V) utilizing an EMIMBF_4_ ionic liquid electrolyte. The symmetrical coin cell supercapacitor device achieved a notable energy density of approximately 23.52 W h kg^−1^ when subjected to an applied current density of 0.66 A g^−1^. Furthermore, Li–S batteries were assembled, incorporating a composite cathode composed of activated carbon derived from biomass and sulfur. Subsequently, cyclic voltammetry alongside charge–discharge assessments at varying scan rates and C-rates were performed, respectively. The sulfur–biomass-derived activated carbon (BAC) composite delivers an initial discharge capacity of 661 mA h g^−1^ at a C-rate of 0.05C. Long-term cycling tests were conducted at 1C and 0.5C over 500 cycles, achieving coulombic efficiencies of approximately 99% and 97%, respectively, in sulfur–biomass-derived activated carbon composite-based Li–S batteries. Hence, our research showcases the scalable synthesis of biomass-derived activated carbon and its utilization as a versatile electrode material, laying the groundwork for the next generation of multifunctional sustainable energy storage systems.

## Abstract 2

The fabrication and electrochemical characterization of multifunctional energy storage devices using biomass-derived activated carbon sourced from indigenous European deciduous trees.

## Graphical abstract

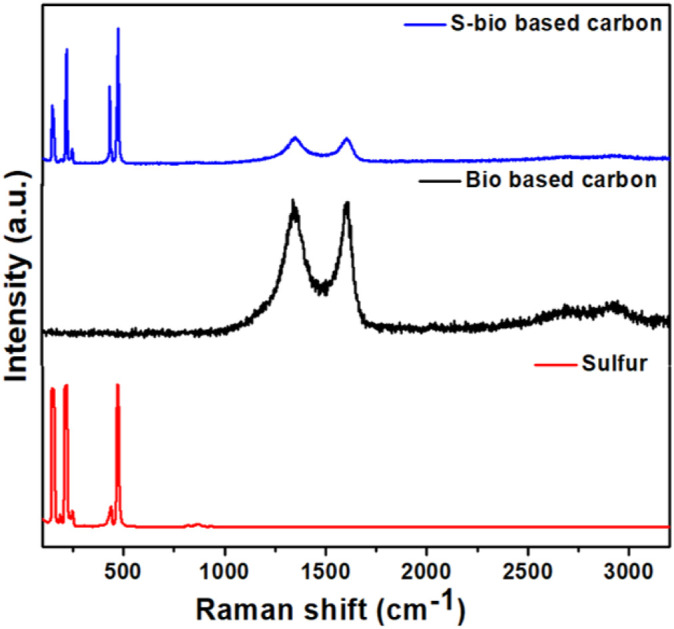

## 1. Introduction

Significant advancements in energy storage technologies have been evident in recent decades, particularly in response to the rising utilization of renewable energy sources. This progress has been particularly notable in tandem with the increased demand for consumer electronics and the advent of electric vehicles (EVs).^1–3^ These developments underscore the imperative for the creation of high-performance energy storage devices. Hence, the research attention has shifted majorly towards energy storage devices such as supercapacitors and lithium–sulfur (Li–S) batteries, due to the growing demand for high-power and high-energy-density energy storage systems in electric vehicles and grid storage applications.^4,5^ Supercapacitors, which complement batteries, are garnering attention across various fields of application. Unlike batteries, supercapacitors exhibit high power capabilities derived from diverse charge storage mechanisms but are constrained by limited energy density. The charge storage mechanisms are commonly classified into double-layer formation occurring at the electrode/electrolyte interface and reversible redox reactions happening across the electrode surface.^6,7^ These processes, characterized by electrical double-layer formation and redox reactions, exhibit high reversibility, leading to both high specific power and prolonged cycle life.^8,9^ Recent research emphasizing increased energy densities underscores the significance of supercapacitors across various applications. Enhancing energy density (*E* = 0.5C V2) in supercapacitors can be accomplished through two main strategies: (i) utilizing ionic or organic electrolytes to broaden the operational potential window of the supercapacitor, extending it up to 2.5–3.5 V. (ii) Improving the specific capacitance or capacity of the electrode to increase overall energy storage capabilities. Hence, supercapacitors employing organic liquid electrolytes offer significantly higher energy density in comparison to those using aqueous electrolytes.^10^

Furthermore, to fulfill the energy requirements of EVs and grid storage systems, there is a pressing need to enhance the energy density of current lithium-ion batteries (LIBs). Nevertheless, LiBs face limitations in large-scale applications because of their lower theoretical capacity and energy density (which presently range from 150 to 300 W h kg^−1^).^11,12^ In recent times, Li–S batteries have garnered consideration as potential substitutes for LiBs in both large-scale applications and portable electronic devices, owing to their superior theoretical capacity (1672 mA h g^−1^) and energy density.^13^ Moreover, sulfur boasts abundant availability, eco-friendliness, non-toxicity, and low cost, rendering it ideal for large-scale applications. Nevertheless, Li–S batteries encounter certain limitations, including low conductivity, significant volume expansion, and the generation of lithium polysulfides. These factors contribute to reduced sulfur utilization and rapid capacity degradation, resulting in poor coulombic efficiency.^14,15^ Numerous approaches have been investigated to address these challenges, including the utilization of novel cathode materials, adjustments to cell components, and the implementation of lithium protection mechanisms. One prevalent method involves incorporating sulfur into the carbon matrix using conductive carbons as cathode materials, facilitating electron transfer.^16,17^

Therefore, a potential solution to tackle the aforementioned challenges involves utilizing waste biomass-derived materials as precursors for producing porous and/or conductive carbon, thereby enhancing the performance of Li–S batteries and supercapacitors.^18,19^ This concept holds great appeal due to its simplicity, low cost, and the abundance of precursor materials availability. Moreover, it facilitates the transition of energy storage devices, specifically Li–S batteries and supercapacitors, from laboratory-scale experimentation to real-world applications in the marketplace. For example, Bongu *et al.* fabricated interconnected porous carbon from Phyllanthus emblica (amla) fruit for utilization in potassium ion batteries and supercapacitors.^20^ The fabricated symmetric supercapacitor using porous carbon obtained a high energy density of 57.5 W h kg^−1^ in an organic electrolyte. Rajesh *et al.* reported the use of pinecone-derived activated carbon for fabricating both symmetric and asymmetric supercapacitors with a 1 M Na_2_SO_4_ aqueous electrolyte.^21^ The resulting symmetric and asymmetric supercapacitor devices achieved energy densities of approximately 16.1 W h kg^−1^ and 8.1 W h kg^−1^, respectively. Subsequently, Vinayagam and colleagues utilized two distinct biomass sources-Syzygium cumini fruit shells (SCFS) and Chrysopogon zizanioides roots (CZR)-to fabricate symmetric supercapacitors, achieving an energy density of around 16.72 W h kg^−1^.^22^ Furthermore, Saini *et al.* published a comprehensive review highlighting various biomass-derived activated carbons for supercapacitor applications.^23^ Their study provided an overview of different biomass sources, including tea leaves, celtuce leaves, dead neem leaves, sunflower seed shells, corn grains, firewood, pistachio shells, rice husk, rice straw, and coconut shells, for the development of supercapacitor devices. Similarly, these biomass-derived materials hold significant potential for battery applications, particularly in Li–S batteries. Likewise, Nema *et al.* and their colleagues documented the application of porous carbon obtained from discarded watermelon rind for use in Li–S battery applications.^24^ The Li–S battery obtained a high initial discharge capacity of 1176 mA h g^−1^ at a 0.5C rate. Półrolniczak *et al.* utilized activated carbon derived from waste mandarin peels to fabricate a Li–S battery cathode.^25^ The synthesized carbon features a hierarchical micro-macroporous structure with a sponge-like network of interconnected microfilaments. The resulting Li–S battery demonstrated an initial specific discharge capacity of 886 mA h g^−1^ at 0.1C. Additionally, Liu and colleagues published a review article highlighting the synthesis and functionality of carbon derived from various biomass sources for Li–S batteries.^26^ Their study explored the electrochemical impact of structural diversity, porosity, and surface heteroatom doping on Li–S battery performance.

Herein, we propose an uncomplicated technique for producing activated carbon derived from European hornbeam through a pyrolysis process, building upon our prior research.^27^ This method aims to harness the material for applications in Li–S batteries and supercapacitors. We constructed both symmetrical coin cell supercapacitor devices and printable miniaturized supercapacitor devices. These devices operated effectively within an operational potential range of 2.5 V (0.0–2.5 V) using an EMIMBF_4_ ionic liquid electrolyte. The symmetrical coin cell supercapacitor device delivered a high energy density of about 23.52 W h kg^−1^ at an applied current density of 0.66 A g^−1^. Additionally, a Li–S battery was constructed utilizing a composite cathode comprised of activated carbon derived from biomass and sulfur. Subsequently, cyclic voltammetry was conducted, along with charge–discharge measurements at different scan rates and C-rates, respectively. The sulfur–BAC composite exhibits an initial discharge capacity of 661 mA h g^−1^ at a C-rate of 0.05C.

## 2. Experimental section

### 2.1 Synthesis of biomass-derived activated carbon (BAC)

Activated carbon was obtained from a mixture of deciduous trees, including Birch, Fagaceae, and Carpinus betulus (commonly known as European hornbeam), through pyrolysis in an air atmosphere at a temperature range of 500–700 °C for 10 minutes. The resulting carbon material was designated as biomass-derived activated carbon (BAC) and was prepared in collaboration with a company ‘BIRKO PROJEKT’ (Zielonka, Poland).

### 2.2 Synthesis of sulfur–BAC composites

A black-colored uniform mixture was obtained by thoroughly blending sulfur powder and BAC in a high-speed planetary mixer, with a predetermined mass ratio of 90% sulfur and 10% BAC. The mixture was heated at 155 °C for 10 hours in an argon atmosphere, employing a tube furnace with a temperature ramp rate of approximately 3 °C per minute. During this process, sulfur began to melt due to its low viscosity, causing it to diffuse into the meso/microporous carbon region of the BAC particles. This resulted in the formation of a sulfur–bio-based carbon composite, impregnated by the molten sulfur.

### 2.3 Instrumentation

Various pivotal material characterization techniques were employed to study the structural and morphological behaviors of the sulfur–BAC composite samples and electrodes prepared, as these factors are crucial in determining the electrochemical performance of the cells. The crystal structures of sulfur, bio-based carbon, and sulfu–BAC were determined using an Empyrean X-ray diffractometer (XRD) instrument manufactured by Malvern Panalytical, UK. Cu-Kα radiation (*λ* = 1.54184 Å) was employed for the analysis.

Laser Raman spectroscopy was conducted on sulfur, bio-based carbon, and sulfur–BAC using the Renishaw Invia Raman spectrometer. The laser excitation source utilized had a wavelength of 514 nm. The infrared spectra were collected using FT-IR spectrometer Nicolet iS50 (Thermo Scientific, Madison, Wisconsin, USA) with a DTGS detector. The following parameters were used for measurement: spectral region 4000–400 cm^−1^, spectral resolution 4 cm^−1^; 64 scans; Happ-Genzel apodization. Treatment of spectrum: polynomial (second order) baseline, subtraction of spectrum of pure potassium bromide. XPS spectra have been obtained using a Phoibos 100 X-ray photoelectron spectrometer operating in Fixed Analyzer Transmission (FAT) mode (SPECS) with a 5-channel MCD-5 detector (SPECS). A monochromatic X-ray source XR50 with FOCUS 500 (SPECS) was used with an Al X-ray tube and Kα line (energy of 1486.6 eV) at 15 kV, 400 W, a flood gun FG 50 (SPECS) was used at 1 V, 1uA settings. The powder sample was placed on a double side carbon conductive adhesive tape in a thick layer of about 1 mm. The electrons were collected in the normal direction, the spectrometer operated in medium area mode with the entrance orbital slit set to 7 × 15 mm^2^, and the exit slit was fully open. The Iris slit was set to 35 mm. For quantitative analysis, we used high-resolution spectra with 10 eV pass energy accumulated in 5 passes. The data were processed in CasaXPS software with the use of a Shirley background and RSF from a built-in database. The surface morphology of BAC powder was investigated using a scanning electron microscope (SEM, CrossBeam Auriga, Carl Zeiss). The Sorptomatic 1990 apparatus (Thermo Electron Corporation, USA) was used to measure the specific surface area of the carbon material. Nitrogen flow was employed, and the surface area was calculated using the Brunauer–Emmett–Teller (BET) adsorption isotherm with the Advance Data Processing software. The Horváth–Kawazoe and Barnett–Joyner–Halenda models were used to calculate the specific surface area of micro- and mesopores in the BAC sample.

### 2.4 Construction of BAC coin cells

BAC coin cells were assembled into a symmetric supercapacitor configuration resembling a coin-cell (CR2032) type setup. The active material (BAC), carbon black, and PVDF were mixed in a weight ratio of 85 : 10 : 5 and thoroughly ground using an NMP dispersant until a homogeneous slurry was obtained. This slurry was then applied onto a stainless-steel (SS) coin cell substrate measuring 15.4 mm × 0.2 mm using a slurry coating technique. Subsequently, the coated substrate was dried overnight in an oven at 70 °C. Two BAC electrodes, considered ideal, were utilized, with filter paper serving as the separator between them. The BAC coin cells were crimped using an electric coin-cell crimping machine sourced from MTI, Korea. The entire fabrication process was conducted within a glove box environment containing less than 0.1 ppm of moisture and oxygen.

### 2.5 Construction of BAC printed device

The fabrication of high-precision interdigitated supercapacitor electrodes (utilizing the above-mentioned prepared slurry) was achieved using the Voltera V one printer. The built-in drawing software (KiCad) was utilized for designing the graphics and specifying the electrode dimensions. Printing took place on a poly(ethylene terephthalate) (PET) substrate from Sigma-Aldrich, which underwent meticulous cleaning with acetone, methanol, and isopropylalcohol (IPA) solvents using an ultrasonic cleaner. Following printing, the electrodes underwent annealing at 60 °C atop a hot plate for 4 hours.

### 2.6 Electrochemical analysis of BAC coin cell

The electrochemical performance assessment of the BAC coin cells involved cyclic voltammetry (CV), galvanostatic charge–discharge (GCD), and electrochemical impedance spectroscopy (EIS) techniques, alongside long-term cyclic stability tests. All electrochemical measurements were conducted utilizing the CHI1600E electrochemical workstation. The ionic liquid 1-ethyl-3-methylimidazolium tetrafluoroborate (EMIMBF_4_, provided by Sigma-Aldrich) serves as the electrolyte in the electrochemical analysis of BAC coin cells and printed devices. The specific capacitance (*C*_sp_), energy density (*E*), and power density (*P*) of the BAC coin cells were determined through the following equations;^10^

1 *C*_sp_ = [(∫*I*d*V*)/(*s* × Δ*V* × *m*)]

2 *C*_sp_ = [(*I* × Δ*t*)/(Δ*V* × *m*)]

3 *E* = (*I* × Δ*t* × Δ*V*)/(7.2 × *m*)

4 *P* = (3.6 × *E*)/Δ*t*

In this context, “*C*_sp_” represents the specific capacitance (F g^−1^), “*I*” denotes the applied current (A), “Δ*V*” signifies the operating potential window, “*s*” stands for the scan rate (mV s^−1^), “Δ*t*” represents the discharge time (s), and “*m*” indicates the total mass of electro-active material (mg) present in both electrodes.

## 3. Results and discussion

**Scheme 1:**
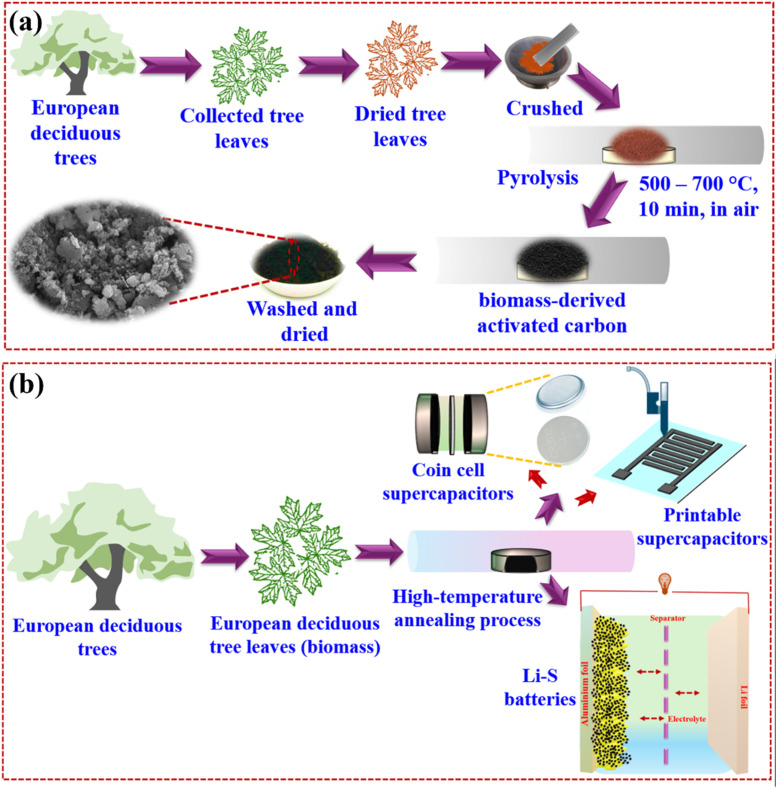
(a) The synthetic diagram for synthesizing BAC from European deciduous tree leaves and (b) fabrication of various supercapacitor devices (coin cell and printable) and Li–S batteries using as-synthesized BAC.

**Fig. 1:**
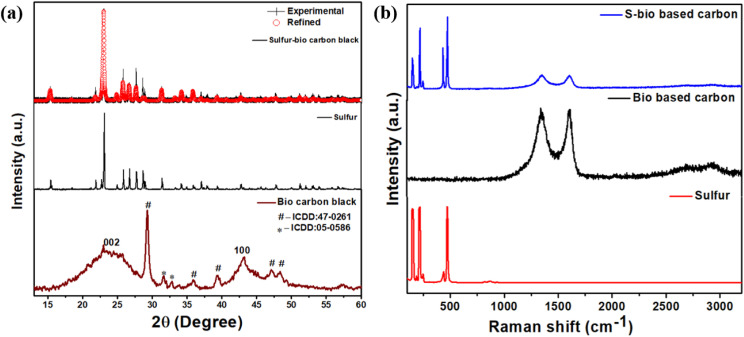
(a) The comparative X-ray diffraction pattern of BAC, sulfur and sulfur–BAC composite. The solid line on the X-ray diffraction (XRD) spectrum depicts the Rietveld-refined representation of the sulfur–BAC composite. (b) The comparative Raman spectra of BAC, sulfur and sulfur–BAC composite.

The BAC utilized in the multifunctional energy storage system was derived from biomass, specifically a blend of deciduous trees like Birch, Fagaceae, and Carpinus betulus (commonly known as European hornbeam). This carbon was produced *via* pyrolysis in an air atmosphere, with temperatures ranging between 500 °C and 700 °C, carried out for a brief duration of only 10 minutes. The resulting BAC was then employed in various energy storage applications, including supercapacitors and Li–S batteries, as illustrated in Scheme 1. Fig. 1(a) presents the comparative X-ray diffraction (XRD) pattern of BAC, sulfur, and melt-impregnated sulfur–BAC composites. The X-ray diffraction (XRD) pattern of BAC reveals broad and faint diffraction peaks around 24° and 43°, corresponding to the graphitic (002) and (100) planes, respectively. These findings closely align with those reported previously.^27^ Furthermore, the XRD pattern of BAC reveals the presence of impurities that can be assigned to the calcium carbonate (calcite, CaCO_3_; ICDD: 05-0586, marked as “*”), as well as sodium calcium hydrogen carbonate phosphate hydrate (Ca_8_H_2_(PO_4_)_6_·H_2_O – NaHCO_3_ – H_2_O; ICDD: 47-0261, marked as “#”) phases. This chemical compound originates from the pyrolysis process (10 minutes at a temperature range of 500–700 °C in an air atmosphere) of carbon materials derived from biomass.^28^ The XRD pattern of sulfur exhibits a close match with the orthorhombic structure (JCPDS no. 77-0145) of sulfur, characterized by the space group *Fddd*, which corresponds to the α-phase of sulfur. Likewise, the XRD pattern of melt-impregnated sulfur–BAC composites indicates minimal deviation from the peak patterns observed in pure sulfur. This suggests that the orthorhombic structure (space group *Fddd*) characteristic of sulfur is retained in the sulfur–BAC composite. The detailed analyses from Rietveld refinement can be found in Table S1.†

Raman spectroscopy measurements were employed to delve deeper into the crystallinity of sulfur, BAC, and melt-impregnated sulfur–BAC composites. The findings from these analyses are illustrated in Fig. 1(b). In the case of pure sulfur, distinct Raman peaks emerge at 153 cm^−1^, 218 cm^−1^, and 472 cm^−1^, each exhibiting subtle shoulders at their base. These peaks correspond to specific vibrational states: 153 cm^−1^ represents the B_3g_ state, indicative of S–S bond bending, while 218 cm^−1^ signifies the B_1g_ state, associated with S–S bond stretching. Furthermore, the peak at 472 cm^−1^ arises from the combination of B_2g_ and B_3g_ states, reflecting a notable presence of S–S bond stretching within the structure.^29,30^ The Raman spectrum of BAC exhibits four distinct bands, situated approximately at 1344 cm^−1^, 1600 cm^−1^, 2688 cm^−1^, and 2933 cm^−1^, which correspond to the D, G, 2D, and D + G bands, respectively, characteristic of typical graphitic materials.^27,31,32^ The Raman spectroscopy analysis of the melt-impregnated sulfur–BAC composite reveals distinct peaks corresponding to sulfur and BAC, situated within low and high-frequency ranges, respectively. These findings were consistently observed at various locations across the samples, confirming the uniform impregnation of sulfur into BAC, thereby facilitating the formation of homogeneous sulfur–BAC composite materials. The comparative Raman spectra of BAC and sulfur–BAC composite (as shown in Fig. 1(b)) illustrate the D and G bands positioned at approximately 1344 cm^−1^ and 1600 cm^−1^, respectively. The *I*_D_/*I*_G_ ratio for BAC is around 1.00, while for the sulfur–BAC composite, it slightly increases to 1.01. This small rise in the *I*_D_/*I*_G_ ratio suggests that the incorporation of sulfur induces a higher degree of structural disorder in the carbon matrix compared to pristine bio-based carbon. As a result, the slight increase in disorder leads to a marginal reduction in electronic conductivity in the sulfur–BAC composite.^33,34^

**Fig. 2:**
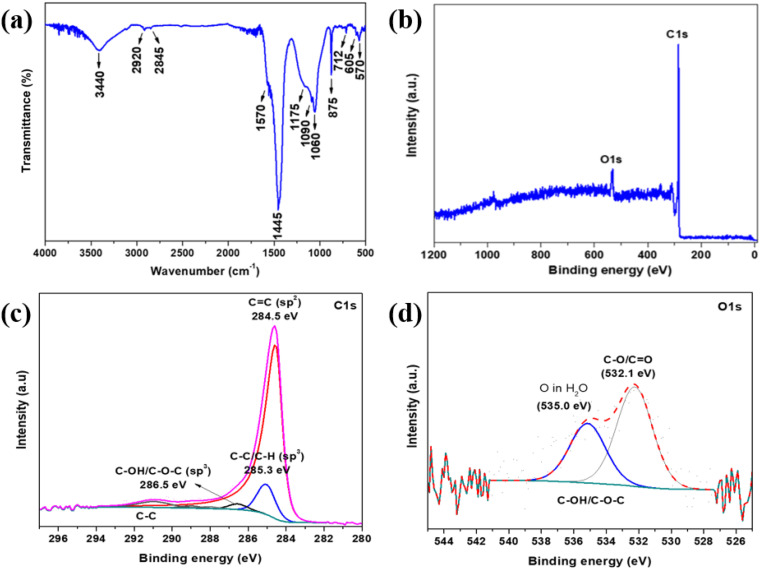
(a) The FTIR spectrum of BAC. The X-ray photoelectron spectroscopy (XPS) of BAC. (b) Survey spectrum. Core level spectrum (c) carbon and (d) oxygen.

The FT-IR analysis (Fig. 2(a)) confirmed the chemical composition of the BAC sample, revealing various functional groups present. The absorption peak at 3440 cm^−1^ indicates O–H bond stretching vibrations from residual water and –OH bonds in phenols, carboxyls, and alcohols attached to graphitic structures. The bands at 2920 cm^−1^ (asymmetrical mode) and 2845 cm^−1^ (symmetrical mode) correspond to CH_2_ stretching vibrations, typical of aliphatic hydrocarbons. Weak bands of the CH_3_ group were also observed. The peak at 1570 cm^−1^ is attributed to aromatic C=C stretching vibrations from polar functional groups like carboxylic acids, lactones, and carboxylic anhydrides. The following peaks at 1445 cm^−1^, 875 cm^−1^, and 712 cm^−1^ represent the vibration bands of carbonates. The broad absorption band at 1175 cm^−1^ can be ascribed to the C–O stretching vibrations, in alcohol, epoxy, or alkoxy groups. The subsequent peaks at 1090 cm^−1^, 1060 cm^−1^, 605 cm^−1^, and 507 cm^−1^ can be assigned to vibration bands of phosphates. Table S2† summarizes the main peaks and their corresponding vibrations identified in the BAC sample.^35,36^ XPS analysis was used to verify the elemental composition of the BAC black sample, as shown in Fig. 2(b–d). The BAC sample consisted of 92.9 at% C and 7.1 at% O. No other elements were detected. The survey spectra (Fig. 2(b)) exhibit two main peaks at binding energies of 536.8 eV and 283.9 eV, which correspond to oxygen and carbon in the sample, respectively. The XPS spectra for carbon (Fig. 2(c)) have been deconvoluted to show several peaks. The peak at 284.5 eV is attributed to the C=C bond (sp^2^), while the peak at 285.3 eV indicates C–C/C–H (sp^3^) bonds. Additionally, the peak at 286.5 eV represents the C–OH/C–O–C (sp^3^) bond and a minor peak at 291.0 eV suggests the π–π bond between C–C. In the O 1s spectra (Fig. 2(d)), two peaks at 532.1 eV and 533.6 eV correspond to the C–O/C=O and C–OH/C–O–C bonds, respectively. Furthermore, a broad peak at 535.0 eV is attributed to adsorbed water on the sample surface. This XPS analysis provides detailed insights into the chemical bonding and elemental composition of the BAC sample, confirming the presence of various carbon and oxygen functional groups.^27,37,38^ FTIR and XPS are complementary analytical techniques that provide valuable information about the chemical composition and bonding present in BAC samples. Furthermore, the carbonates and phosphates group detected through XRD and FTIR analyses were not found in the XPS analysis. This suggests that these functional groups are not present on the surface but rather located within the porous framework.

**Fig. 3:**
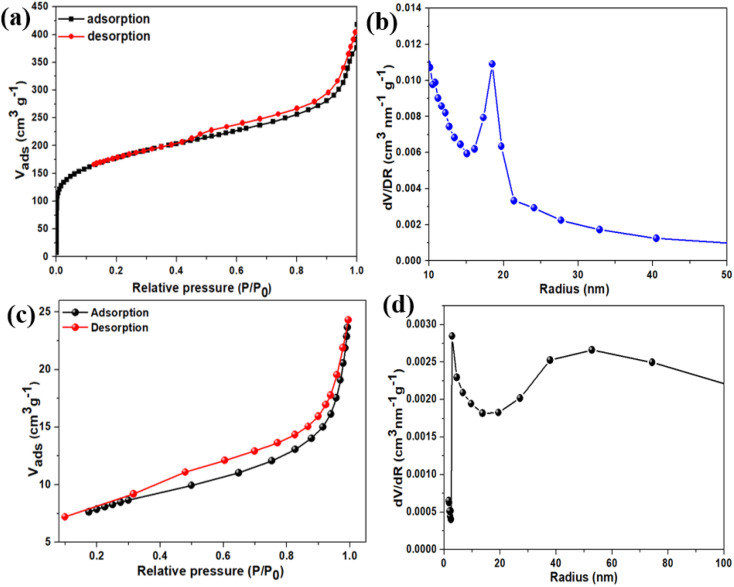
(a) The N_2_ adsorption–desorption isotherm and (b) pore size distribution of BAC. (c) The N_2_ adsorption–desorption isotherm and (d) pore size distribution of sulfur–BAC composite.

The N_2_-absorption–desorption experiments were conducted to further understand the pore structure and measure the specific surface area (SSA) of the BAC sample. The results, presented in Fig. 3(a), show Type II isotherms with an H4 hysteresis loop, indicating the presence of both micro- and mesopores in the material.^39^ The estimated specific surface area was found to be 616 m^2^ g^−1^, highlighting the material's high surface area and potential for various applications requiring high surface area electrodes. The Horváth–Kawazoe model was used to calculate the pore size of the BAC sample, which was found to be approximately 5 nm. This pore size is ideal for the intended supercapacitor application, as it provides optimal surface area and ion accessibility for energy storage. The pore size distribution curve for the BAC sample is shown in Fig. 3(b), providing insights into the distribution of pore sizes within the material. Moreover, the BAC sample's total pore volume (*V*_total_) was measured to be 0.564 cm^3^ g^−1^, with the micropore volume (*V*_micro_) calculated at 0.274 cm^3^ g^−1^. Similarly, the N_2_ adsorption–desorption isotherms and pore size distribution of the sulfur–BAC composite are provided in Fig. 3(c and d), respectively. The BET-specific surface area of the sulfur–BAC composite was found to be 26.64 m^2^ g^−1^ and the pore volume is around 0.031 cm^3^ g^−1^. The significant reduction in surface area and pore volume suggests effective encapsulation and uniform dispersion of sulfur within the pores of BAC. This decrease can be primarily attributed to pore blocking, where sulfur deposits inside the pores, limiting their accessibility. Furthermore, sulfur infiltration may induce structural stress, causing pore shrinkage or collapse. Sulfur agglomeration on the BAC surface can also occupy external pores and active sites, further reducing the available surface area. Similar trends have also been observed in previously published articles.^40–42^ These measurements confirm the material's porous nature and its potential suitability for supercapacitor as well as Li–S batteries applications, as the significant volume of micropores allows for electrolyte penetration. Additionally, the thermogravimetric analysis (TGA) of the melt-impregnated sulfur–BAC composite char is illustrated in Fig. S1 of the ESI.† The figure indicates that approximately 90% of the composite's weight is lost when the sample is heated to around 450 °C. This significant weight loss confirms the presence of approximately 90 wt% sulfur in the sulfur–BAC composite char.

**Fig. 4:**
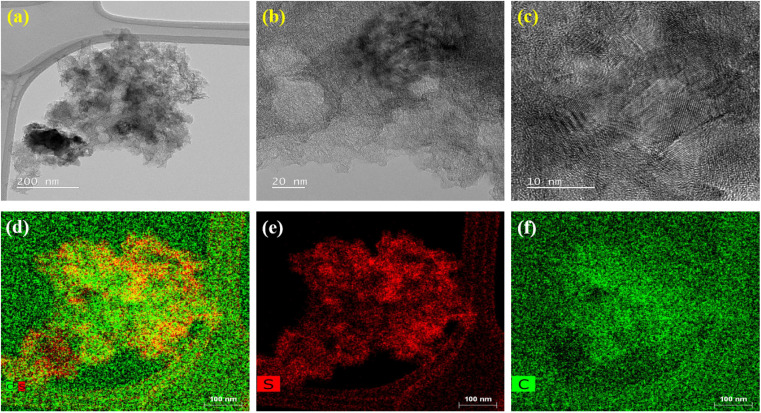
The morphological analysis of melt-impregnated sulfur–BAC composite char is carried out through high-resolution transmission electron microscopy (HR-TEM). (a–c) The HR-TEM micrograph mapping of melt-impregnated sulfur–BAC composite char at various magnifications. (d–f) The HR-TEM elemental mapping of melt-impregnated sulfur–BAC composite char indicates the presence of sulfur and carbon.

The morphology of BAC sample was examined using SEM and TEM analysis, as illustrated in Fig. S2(a–f).† The SEM micrograph revealed a porous structure with particle sizes ranging from 50 to 200 nm. The particles tend to agglomerate, forming larger structures exceeding 1 μm in size. Similarly, the high-resolution TEM micrographs (Fig. S2(d and e)†) reveal a widespread distribution of nanometer-sized particles and pores. Fig. S2(f)† illustrates the TEM mapping of BAC, indicating the presence of carbon nanoparticles. This porous morphology makes the carbon material suitable for applications as an electrode material in supercapacitors or Li–S batteries. Fig. 4(a–f) presents the HRTEM micrographs of a melt-impregnated sulfur–BAC composite char. These micrographs provide high-resolution images showing individual carbon black particles fully coating the sulfur layer and/or microparticles, indicating sulfur diffusion into the interparticle pore regions of the nanocarbon particles. The TEM mapping clearly shows that the carbon black is uniformly distributed on the sulfur microparticles. The SEM micrographs of melt-impregnated sulfur–BAC composite char at various magnifications are presented in Fig. S3(a–c).† These images are consistent with the TEM micrographs. To assess the quality of the coating of the sulfur–BAC composite slurry on aluminum foil, SEM micrographs are shown in Fig. S4(a and b).† These images demonstrate a uniform coating of the sulfur–BAC composite slurry on the aluminum foil for the fabrication of the cathode. This uniform coating of sulfur–BAC is crucial for the stability and reliability of Li–S batteries, helping to mitigate the degradation mechanisms.

### 3.1 Electrochemical characterization

**Fig. 5:**
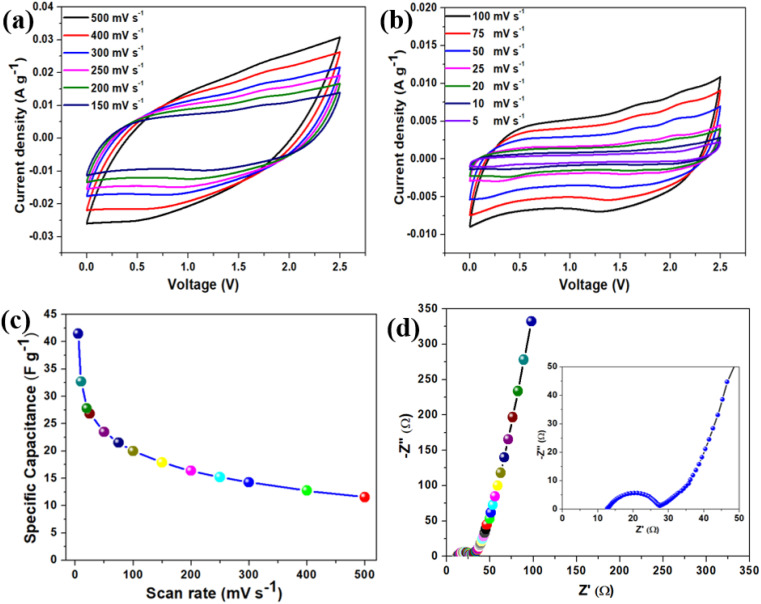
The electrochemical characterization of BAC-based SCSD. (a and b) The cyclic voltammetry profile of BAC-based SCSD was measured at various scan rates (5–500 mV s^−1^). (c) Effect of the gravimetric specific capacitance of BAC-based SCSD with respect to scan rates. (d) The Nyquist plot of BAC-based SCSD and the inset shows the enlarged view of the Nyquist plot.

After detailed physical characterization of the BAC, electrochemical measurements were carried out by fabricating a symmetrical coin cell supercapacitor device (SCSD) using an EMIMBF_4_ ionic liquid electrolyte. The operating potential window of the fabricated SCSD was evaluated in Fig. S5(a),† which indicates that the SCSD worked in the potential window of 2.5 V (0.0–2.5 V) without encountering any indications of evolution. Fig. S5(b)† illustrates the relationship between specific device capacitance and different operating potential windows of SCSD and finds a linear increase in specific capacitance as the operating potential window increases, in agreement with the power law.^43^ The cyclic voltammetry profile of the fabricated SCSD is shown in Fig. 5(a and b), which indicates that the SCSD was recorded at both high (150–500 mV s^−1^) and low (5–100 mV s^−1^) scan rates. In both high and low scan rates, the BAC-based SCSD exhibits typical quasi-rectangular behavior, which can be classified as a type A curve, suggesting characteristics akin to an Electric Double-Layer Capacitor (EDLC).^44^ It is visible that as the scan rate increases, more charge can be stored and released during each potential sweep rate, resulting in larger current peaks, which can be attributed to the capacitive behavior of electroactive materials.^45^ The plot for gravimetric-specific capacitance concerning scan rates is provided in Fig. 5(c), which indicates that the BAC-based SCSD obtained the highest specific capacitance of 41.47 F g^−1^ at a scan rate of 5 mV s^−1^. Moreover, the BAC-based SCSD indicates that even as the scan rate increases significantly by 100 times (from 5 mV s^−1^ to 500 mV s^−1^), the device still maintains the specific capacitance of 11.55 F g^−1^ at a scan rate of 500 mV s^−1^, suggesting that the SCSD exhibits good rate capability. To understand the charge-storage properties and capacitive nature of the fabricated SCSD, electrochemical impedance spectroscopy (EIS) measurements were performed, and the Nyquist plot of SCSD is provided in Fig. 5(d) (with the inset showing the enlarged view). The Nyquist plot consists of a semi-circle (in the high-frequency region) arc followed by a straight line (in the low-frequency region), suggesting a combination of charge transfer processes and diffusive transport within the system.^46^ From the Nyquist plot, it is found that the equivalent series resistance (ESR) and charge-transfer resistance (*R*_ct_) of the BAC-based SCSD are about 12.34 Ω and 14.94 Ω, respectively. The straight line (Warburg line) arises in the low-frequency region due to the Warburg impedance, which is a characteristic of diffusional processes within the electrolyte or the bulk of the electrode material.^47^ The Bode phase angle plot (as shown in Fig. S6(a)†) of BAC-based SCSD is approximately around −75°, indicating the capacitive behavior of BAC. To find out the specific device capacitance of BAC-based SCSD varies with frequency from the EIS spectrum, a plot of specific capacitance *vs.* frequency is provided in Fig. S6(b)† and found that at a low frequency of 0.01 Hz, the specific device capacitance is around of 16 F g^−1^.

**Fig. 6:**
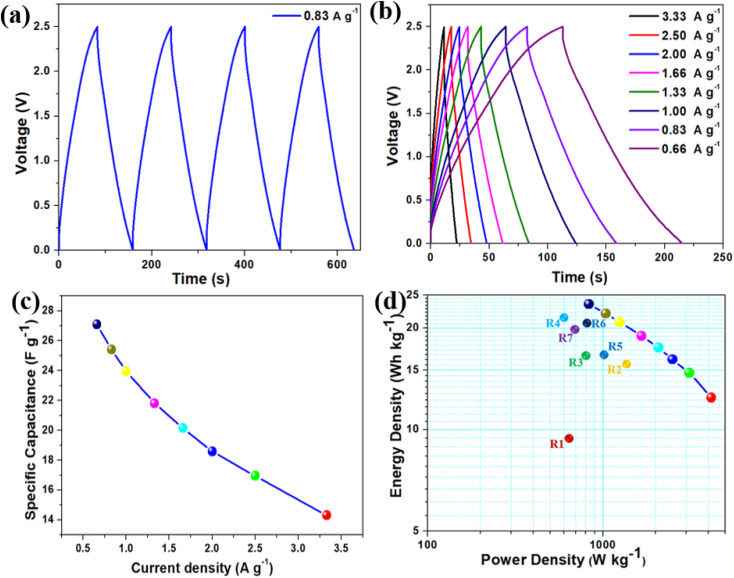
(a) The galvanostatic charge–discharge profiles of BAC-based SCSD measured at a constant current of 0.83 A g^−1^ in the operating potential window from 0.0 to 2.5 V. (b) Charge–discharge profiles of BAC-based SCSD recorded in different applied current ranges from 3.33 to 0.66 A g^−1^. (c) The effect of discharge current on the gravimetric specific capacitance of BAC-based SCSD, (d) a Ragone plot showcasing the performance metrics of BAC-based SCSD highlights their superior capabilities compared to previously reported supercapacitors utilizing ionic liquid electrolytes. The references (R1–R7) in Fig. 4(D) are provided in Table S3 (in ESI).†

Fig. 6(a) represents the galvanostatic charge–discharge (GCD) profile of BAC-based SCSD at a constant applied current density of 0.83 A g^−1^ in the operating potential window of 2.5 V. The GCD profile exhibits almost symmetric behavior during both the charging and discharging phases, which aligns well with CV behavior. The GCD profile of BAC-based SCSD at various applied current densities (from 3.33 A g^−1^ to 0.66 A g^−1^) is provided in Fig. 6(b), which reveals that the symmetric triangular shape of the GCD profiles is a characteristic behavior associated with the charge storage mechanism attributed to double-layer capacitance.^48^ The influence of applied discharge current on the gravimetric specific capacitance of BAC-based SCSD is shown in Fig. 6(c). The BAC-based SCSD obtained the highest specific capacitance of around 27.09 F g^−1^ at an applied current density of 0.66 A g^−1^. The effect of areal-specific capacitance concerning scan rates and applied currents from CV and GCD profiles is provided in Fig. S7(a) and (b),† respectively. The maximum areal-specific capacitance was found to be approximately 69.11 mF cm^−2^ (at a scan rate of 5 mV s^−1^) and 45.16 mF cm^−2^ (at an applied current density of 0.66 A g^−1^) based on the CV and GCD profiles. The Ragone plot is a common way to visualize the trade-off between energy density and power density for supercapacitor devices and Fig. 6(d) represents the Ragone plot for BAC-based SCSD. The Ragone plot indicates that the BAC-based SCSD delivers a high energy density of about 23.52 W h kg^−1^, accompanied by a corresponding power density of about 833 W kg^−1^ (at an applied current density of 0.66 A g^−1^). The BAC-based SCSD obtained the highest power density, 4166 W kg^−1^, with an increase in the applied current density of around 3.33 A g^−1^ (5 times). The comparative analysis of energy and power density of BAC-based SCSD with other supercapacitor devices is shown in the Ragone plot (Fig. 6(d)) and Table S3.† To prove the durability and reliability of BAC-based SCSD, especially because of practical applications where they may undergo frequent charge and discharge cycles over an extended period, a cyclic stability test was carried out at an applied current density of 3.33 A g^−1^ over 5000 cycles (as shown in Fig. S8(a)†). The BAC-based SCSD showed a retention of around 89% of the initial specific capacitance over 5000 cycles. To verify the decrease in the capacitance retention over 5000 cycles, an EIS measurement was carried out after the cyclic stability test, and the comparative Nyquist plot before and after the cyclic stability test is shown in Fig. S8(b).† It is displayed that after the cyclic stability test, the ESR increases from 12.34 Ω to 16.03 Ω and the *R*_ct_ increases from 14.94 Ω to 19.18 Ω, which could indeed be a reason for the observed capacitance decay over prolonged cycles.^49^

**Fig. 7:**
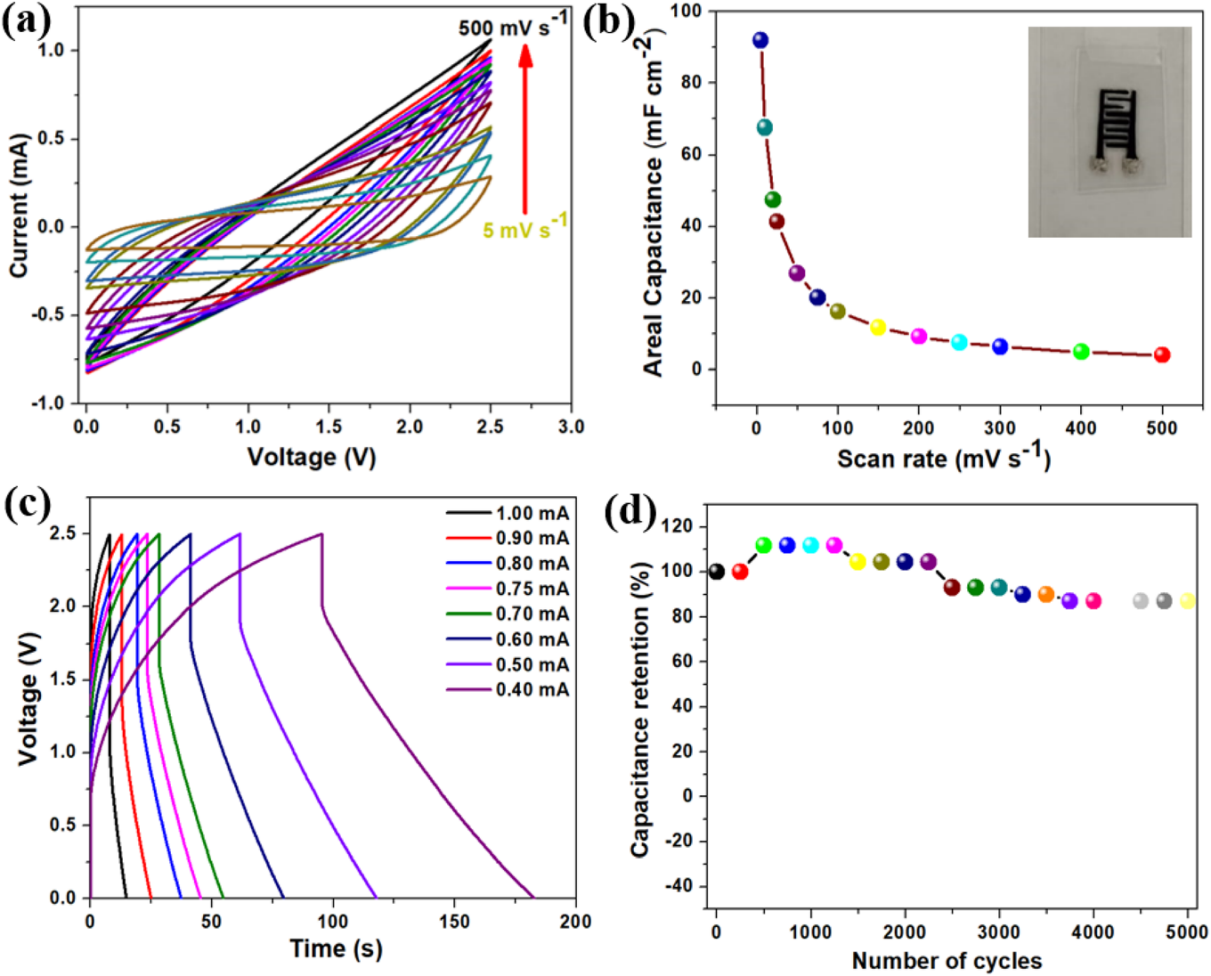
The electrochemical characterization of BAC-based printable device. (a) The cyclic voltammetry profile of the BAC-based printable device was measured at various scan rates (5–500 mV s^−1^). (b) Effect of areal-specific capacitance of BAC-based printable device with respect to scan rates. (c) Charge–discharge profile of BAC-based printable device recorded in different applied current ranges from 1 to 0.40 mA. (d) The cyclic stability performance for the BAC-based printable device over 5000 cycles.

Moreover, a series of electrochemical tests were conducted in a two-electrode configuration to assess the energy storage capabilities of printed BAC-based interdigitated electrodes (IDE) and EMIMBF_4_ ionic liquid electrolyte. These tests aimed to evaluate the supercapacitive performance of the printed BAC-based devices. The cyclic voltammetry profile of printed BAC-based devices is shown in Fig. 7(a) and measurements were performed at various sweep rates (5–500 mV s^−1^) over a working potential window of 2.5 V. At a low scan rate (5 mV s^−1^), the quasi-rectangular voltammograms are evident, indicating that the predominant energy storage mechanism is capacitive due to the formation of electric double layer.^50^Fig. 7(b) demonstrates the impact of sweep rate on the areal capacitance of the printed BAC-based device. Notably, the graph reveals that at a low sweep rate of 5 mV s^−1^, a remarkable areal capacitance of 91.97 mF cm^−2^ was achieved. The digital image of the printed BAC-based device is shown in the inset of Fig. 7(b). Fig. 7(c) presents the galvanostatic charge–discharge (GCD) profile of printed BAC-based devices across a range of applied currents, from 1 to 0.4 mA. The graph illustrates a symmetric triangular shape in the GCD profile. It is observed that as the applied current increases, the charge–discharge profiles exhibit a more rapid behavior at higher current ranges, and conversely, a slower behavior at lower current ranges.^51^ Furthermore, the continuous GCD profiles of printed BAC-based devices were recorded over four consecutive cycles at a constant applied current of 0.75 mA, as depicted in Fig. S9(a).† These profiles reveal a triangular shape with pronounced symmetry across successive cycles, indicating stable and reproducible performance.^51^ Fig. S9(b)† illustrates the impact of applied current ranges on the areal capacitance of printed supercapacitor devices utilizing BAC as the electroactive material. The printed supercapacitor device obtained the highest areal capacitance, around 60.13 mF cm^−2^ at an applied discharge current of 0.4 mA. The Ragone plot, which describes the specific energy and specific power of printed BAC-based devices obtained from the GCD profile is shown in Fig. S10(a).† The printed BAC-based devices delivered an energy density of 0.052 mW h cm^−2^ with the corresponding power density of 2.14 mW h cm^−2^ at an applied current of 4 mA. To assess the long-term cyclic stability of the printed BAC-based device, a continuous GCD test was conducted. The test involved applying a constant current of 0.75 mA over 5000 cycles, as illustrated in Fig. 7(d), and exhibited a capacitance retention of around 86.90% over 5000 cycles. Further examinations were conducted to elucidate the decay in the capacitance retention over 5000 cycles, using electrochemical impedance spectroscopy. The Nyquist plot of printed BAC-based devices is provided in Fig. S10(b).† It is evident from the Nyquist plot that following the cyclic stability test, the equivalent series resistance (ESR) rose from 547 Ω to 815 Ω, while the charge transfer resistance (*R*_ct_) increased from 686 Ω to 910 Ω. This increase could potentially explain the observed decay in capacitance over 5000 cycles.^49^

### 3.2 Electrochemical characterization of Li–S battery

**Fig. 8:**
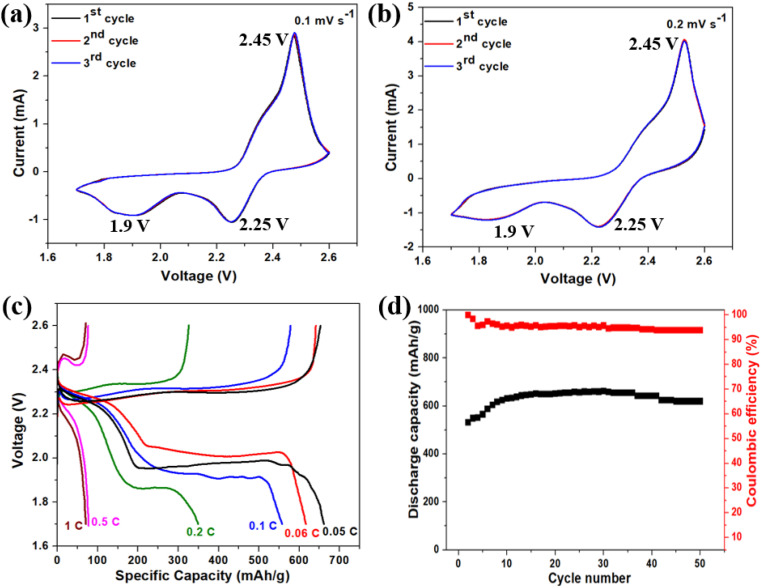
The electrochemical characterization of sulfur–BAC composite-based cathode. The cyclic voltammetry profile of sulfur–BAC composite-based cathode at a scan rate of (a) 0.1 mV s^−1^ and (b) 0.2 mV s^−1^. (c) The charge–discharge sulfur–BAC composite-based cathodes at various C-rates (0.05–1C). (d) The cyclic stability and coulombic efficiency of sulfur–BAC composite-based cathode over 50 cycles.

The cyclic voltammetry (CV) measurements and charge–discharge (GCD) profiles were utilized to investigate the electrochemical characteristics and reversibility of sulfur–BAC composite cathodes in the potential range of 1.75 to 2.6 V. Fig. 8(a–b) shows the CV profile of the sulfur–BAC composite cathode at a constant scan rate of 0.1 mV s^−1^ and 0.2 mV s^−1^ over three continuous cycles. Both the CV profiles (0.1 mV s^−1^ and 0.2 mV s^−1^) indicate that two distinct reduction peaks appearing at approximately 2.25 and 1.9 volts are attributed to the sequential reduction process of elemental sulfur. Initially, elemental sulfur is reduced to soluble lithium polysulfides (Li_2_S_*n*_, where *n* ranges from 4 to 8). Subsequently, these lithium polysulfides undergo further transformation into insoluble Li_2_S_*n*_ (where *n* equals 1 or 2). Throughout the anodic scan, a prominent singular peak emerges at a potential of 2.45 V, indicating the oxidation of Li_2_S back into elemental sulfur chains.^52,53^ Noticeably, there are only minimal variations in the CV peak at both the scan rates of sulfur–BAC composite cathodes in successive cycles, affirming the high electrochemical reversibility and outstanding stability of the cathode.^54^ To conduct further assessment of lithium storage characteristics, galvanostatic charge–discharge (GCD) studies were performed on the Li–S battery incorporating the sulfur–BAC composite cathode. Fig. 8(c) represents the GCD profile of fabricated Li–S batteries recorded at various C-rates (from 0.05C to 1C). The GCD profile displays two distinct plateau potentials during discharging, which represent the primary features indicating the evident transformation of solid sulfur into higher-order polysulfides through reaction with Li^+^ ions, and subsequent conversion of polysulfides into lower-order Li_2_S. A distinct, solitary plateau potential emerges around 2.3 V during the charging process, attributed to the development of elemental sulfur chains. The GCD profiles substantially confirmed the findings of the CV results and were well aligned with previous reports.^53^ At a C-rate of 0.05C, the Li–S battery equipped with a sulfur–BAC composite cathode demonstrates an initial discharge capacity of 661 mA h g^−1^ and a corresponding charging capacity of 653 mA h g^−1^. From Fig. 8(c), it is demonstrated that, as the C-rate increases, there is a clear trend of decreasing discharge capacity. This decline is attributed to the limited mobility of electrolyte ions at higher current densities, leading to diminished penetration into the electrode material and consequently reducing the effective utilization of the active material.^52^ To delve deeper into analyzing the sulfur–BAC composite-based cathode performance, the cycling stability and coulombic efficiency of the Li–S battery were assessed at a 0.06C rate over continuous 50 cycles. The resultant data is depicted in Fig. 8(d). The Li–S battery, incorporating the sulfur–BAC composite-based cathode, demonstrates exceptional cycling stability across 50 cycles, retaining 93.89% coulombic efficiency. This cyclic stability is reflected in its consistent discharge capacity, reaching from 531 mA h g^−1^ to 654 mA h g^−1^ after 30 cycles. Moreover, the cyclic stability performance of sulfur–BAC composite-based cathode was performed over 500 cycles at 1C and 0.5C and shown in Fig. S11(a and b),† respectively. The capacity increases from 53 mA h g^−1^ to 114 mA h g^−1^ and the coulombic efficiency was obtained at around 99% over 500 cycles at 1C. Similarly, at 0.5C, the capacity increases from 70 mA h g^−1^ to 153 mA h g^−1^ and the coulombic efficiency was obtained at around 97% over 500 cycles. Table S4† represents the comparative analysis of different sulfur–carbon composite cathodes and the S–BAC composite-based cathode.

## Conclusions

BAC was efficiently synthesized through a straightforward pyrolysis process using European deciduous trees. Structural and morphological analyses employing XRD, FTIR, Raman spectroscopy, XPS, BET, SEM, and TEM techniques validated the creation and quality of BAC. Both BAC (as electroactive material) and EMIMBF_4_ ionic liquid (as electrolyte) were utilized in fabricating coin-cell supercapacitors and printed miniaturized supercapacitor devices. The BAC-based symmetric supercapacitor device obtained an energy density of approximately 23.52 W h kg^−1^ at an applied current density of 0.66 A g^−1^ with excellent capacitance retention of around 89% of the initial specific capacitance over 5000 cycles. Under a C-rate of 0.05C, the Li–S battery featuring a cathode composed of sulfur–BAC composite exhibits an initial discharge capacity of 661 mA h g^−1^. Thus, our research suggests that the pyrolysis process presents a novel and promising approach for manufacturing BAC material that meets the essential criteria for serving as an electrode material in multifunctional energy storage applications. Additionally, it's noteworthy to mention that the proposed synthesis method is highly scalable, cost-effective, and well-suited for commercial implementation.

## Data availability

The data supporting this article have been included as part of the article or its ESI file.†

## Conflicts of interest

There are no conflicts to declare.

## Supplementary Material

NA-OLF-D5NA00018A-s001
