# Supplementary material for: A renewable carbon material derived from native European deciduous trees serves as a sustainable electroactive substance for multifunctional energy storage systems
Source: Nanoscale Adv. 2025 Apr 14;7(11):3331–43. doi: 10.1039/d5na00018a (PMC11995164; doi:10.1039/d5na00018a)
Supplement: NA-007-D5NA00018A-s001 [file NA-007-D5NA00018A-s001.pdf]

Supplementary Information (SI) for Nanoscale Advances.
This journal is © The Royal Society of Chemistry 2025

Supplementary information

A renewable carbon material derived from native European deciduous trees serves as a sustainable electroactive substance for multifunctional energy storage systems

Surjit Sahoo[1,2*], Thiba Nagaraja[1], Monika Michalska[4] , Suprem R. Das [1, 3*]

[1]Department of Industrial and Manufacturing Systems Engineering, Kansas State University, Manhattan, KS 66506, USA

[2]Mechanical Engineering Department, Indian Institute of Technology, Jammu 181221, Jammu & Kashmir, India.

[3]Department of Electrical and Computer Engineering, Kansas State University, Manhattan, KS 66506, USA

[4]Department of Chemistry and Physico-Chemical Processes, VSB-Technical University of Ostrava, 17. listopadu 2172/15, 70800 Ostrava-Poruba, Czech Republic

* Corresponding author, Email: srdas@ksu.edu, surjit488@gmail.com

Table S1. Refinement of X-ray Rietveld parameters for a composite of sulfur and BAC.

Structure (S-BAC)	Space group	Density of S (g/cc)	Lattice parameter	Site	x	y	z
Orthorhombic	Fddd	2.0702357818002133	a = 10.4751215 Å, b = 12.878142 Å, c = 24.50939 Å and α = β = γ = 90°	S1	0.81294745	-0.037023615	-0.050928295
				S2	0.7427133	-0.0037920221	0.017941648
				S3	0.7765132	0.042594533	0.07237818
				S4	0.78624827	-0.09246102	0.1292379

Table S2. Assignment of main peaks and their corresponding vibrations identified in the BAC sample.

Wavenumber	Assignment
3440 cm^{-1}	stretching vibration O-H<br>residual water as moisture, a band of a functional group -OH bond on a graphitic structure
2920 cm^{-1}<br>2845 cm^{-1}	stretching vibration CH_2 *(2920 cm^{-1} asymmetrical mode, 2845 cm^{-1} symmetrical mode)*<br>typical band of aliphatic hydrocarbons; bands of the CH_3 group are also visible in the spectrum
1570 cm^{-1}	stretching vibration C=C
1445 cm^{-1}<br>875 cm^{-1}<br>712 cm^{-1}	vibration bands of carbonates
1175 cm^{-1}	stretching vibration C-O<br>a band of alcohol, epoxy or alkoxy groups
1090 cm^{-1}<br>1060 cm^{-1}<br>605 cm^{-1}<br>507 cm^{-1}	vibration bands of phosphates

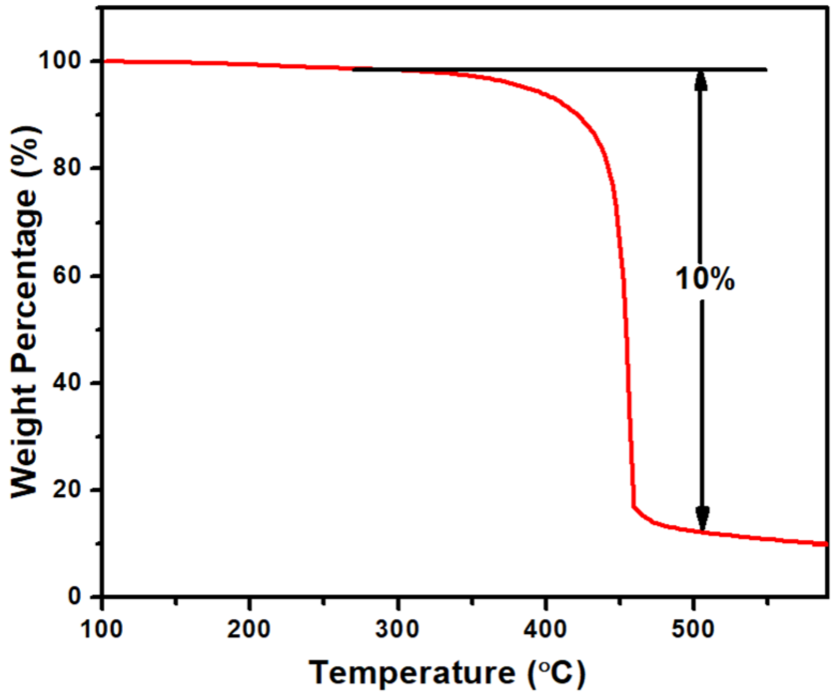

Figure S1. A thermogravimetric analysis (TGA) plot of the melt-impregnated sulfur-BAC composite char reveals that the sample contains ~90 wt% sulfur.

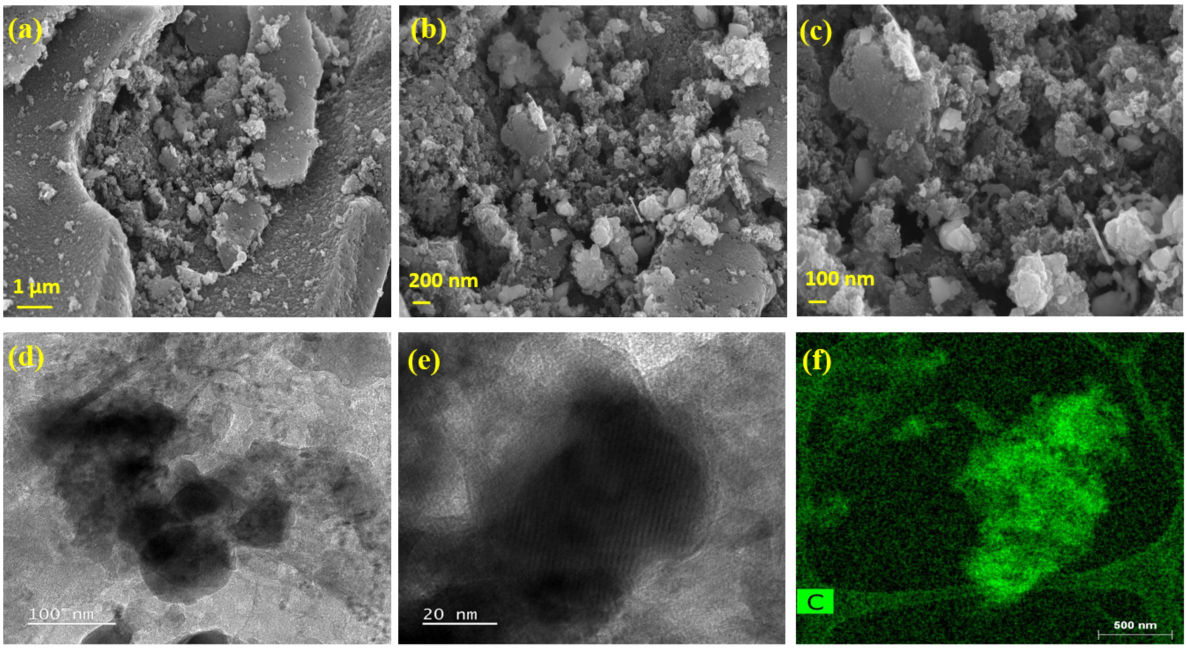

Figure S2. The morphological analysis of BAC is carried out through Field emission scanning electron microscopy (FE-SEM) and High-resolution transmission electron microscopy (HR-TEM). (a-c) The FE-SEM micrograph of BAC at various sizes. (d and e) The HR-TEM micrograph mapping of BAC at various magnifications. (f) The HR-TEM elemental mapping of BAC indicates the presence of carbon.

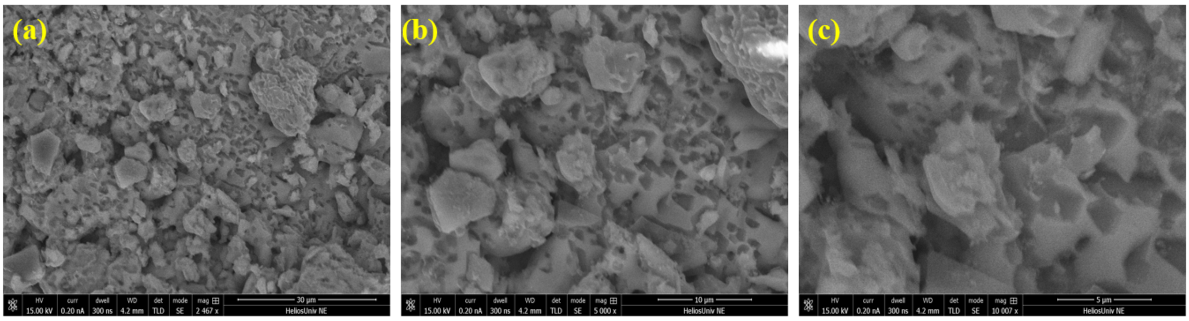

Figure S3. The FE-SEM micrograph of melt-impregnated sulfur-BAC composite char at various magnifications.

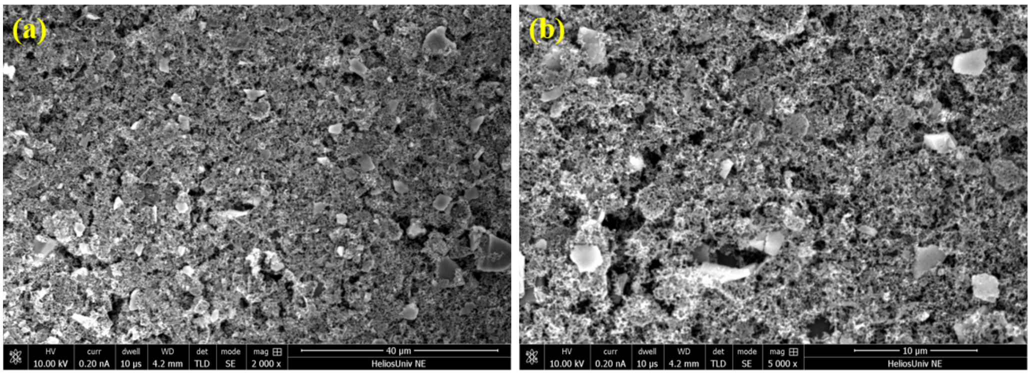

Figure S4. The FE-SEM micrograph of coated sulfur-BAC composite slurry on aluminum foil at various magnifications.

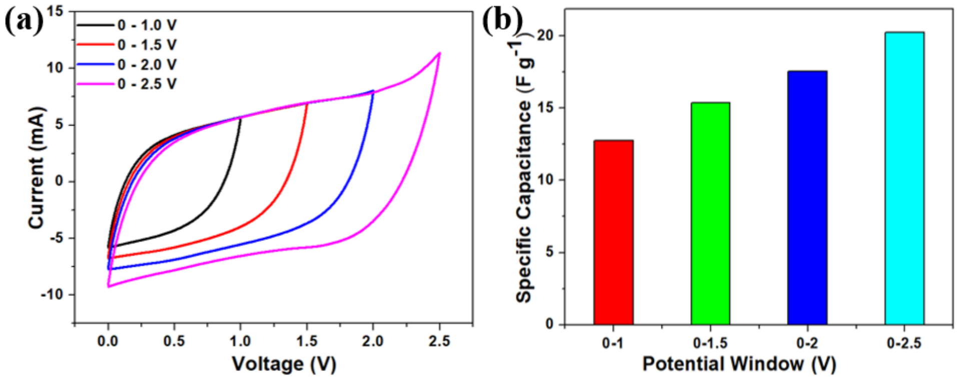

Figure S5. (a) The cyclic voltammetry profiles of the BAC-based SCSD were measured at the different operating potential windows (1.0 to 2.5 V). (b) The Effect of specific capacitance of BAC-based SCSD with respect to the different operating potential windows (1.0 to 2.5 V).

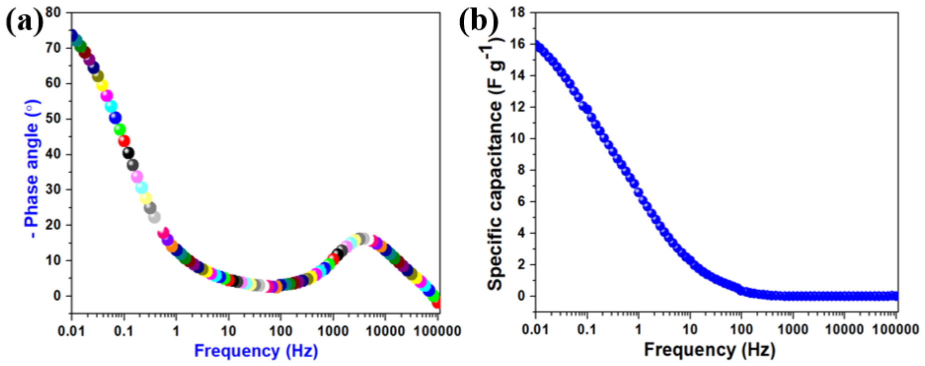

Figure S6. (a) The Bode phase angle plot for BAC-based SCSD. (b) The plot of a specific capacitance with respect to frequencies of the BAC-based SCSD.

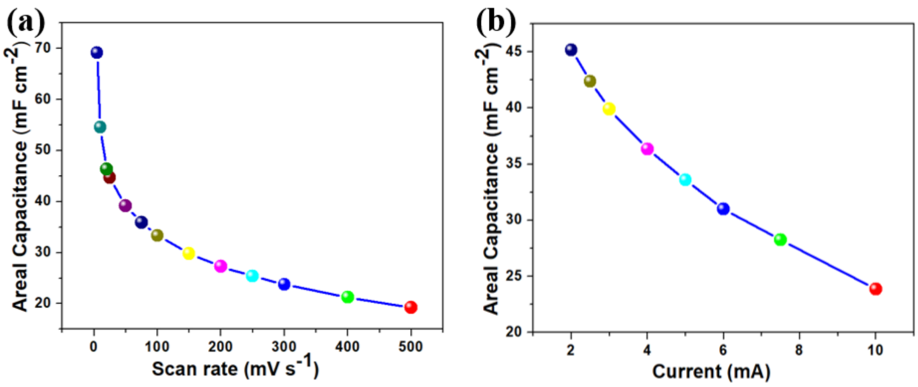

Figure S7. The plot of (a) areal capacitance vs. scan rate and (b) areal capacitance vs. applied current of the BAC-based SCSD.

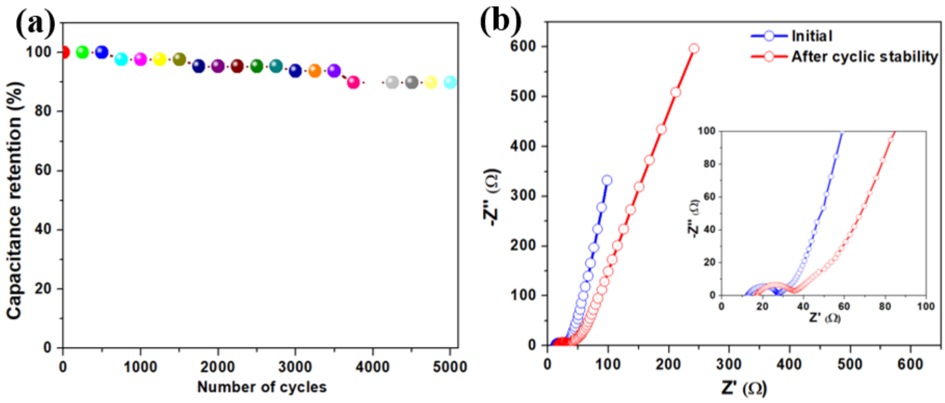

Figure S8. (a) The cyclic stability performance for the BAC-based SCSD over 5000 cycles. (b) The Nyquist plot before and after cyclic stability of the BAC-based SCSD.

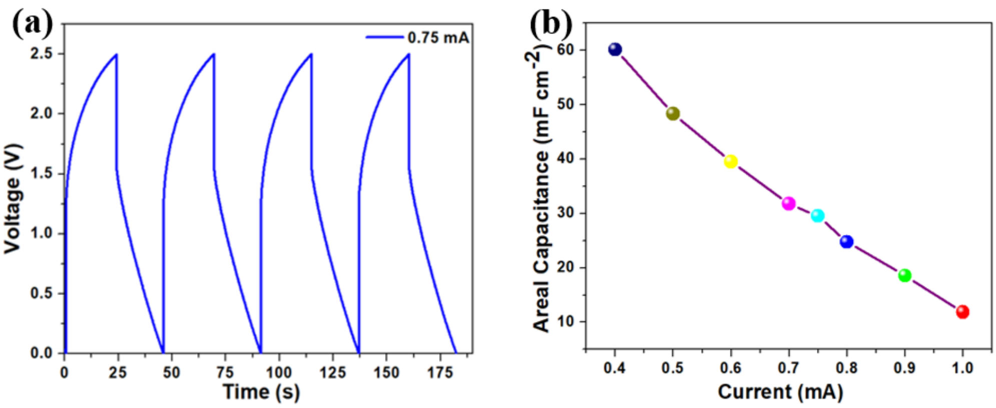

Figure S9. (a) The galvanostatic charge-discharge profile of the BAC-based printable device was measured at a constant current of 0.75 mA in the operating potential window from 0.0 to 2.5 V. (b) The plot of areal capacitance vs. applied current of the BAC-based printable device.

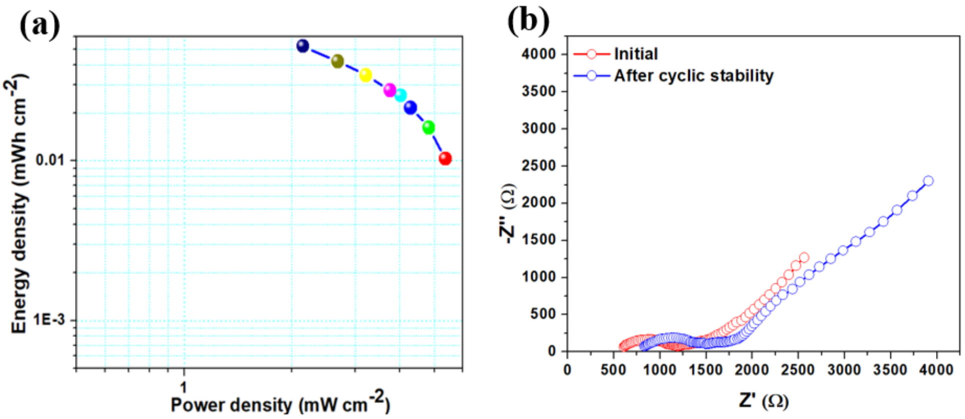

Figure S10. (a) The Ragone plot of the BAC-based printable device. (b) The Nyquist plot of the BAC-based printable device before and after cyclic stability.

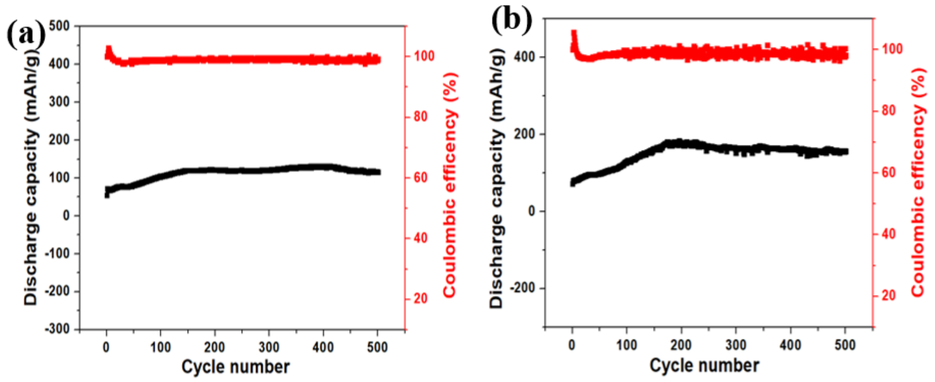

Figure S11. The cyclic stability and Coulombic efficiency of sulfur-BAC composite-based cathode over 500 cycles at a C-rate of (a) 1 C and (b) 0.5 C, respectively.

Table S3. Summary of electrochemical performances of BAC-based SCSD and recently reported supercapacitor devices using ionic liquid and organic liquid as electrolytes.

Si. no.	Electrode material	Electrolyte	Potential window (V)	Energy density (Wh kg^{-1})	Power density (W kg^{-1})	Reference
1	rGO	$LiClO_4$/PC	1.6	9.4	678	R1[1]
2	rGO	$BMIBF_4$	4	16.5	1600	R2[2]
3	rGO	[SET_3][TFSI]-GO	2.5	17.7	875	R3[3]
4	rGO-CMK-5	$LiPF_6$	2.5	23.1	-	R4[4]
5	Carbon	$EMIMBF_4$-$EMIMTf_2N$	4	17	1000	R5[5]
6	Carbon-grafted NiO	EMI-DCA	4	21	-	R6[6]
7	Activated carbon	$PYR_{14}TFSI$	3.5	20	700	R7[7]
**8**	**biomass-derived activated carbon**	**$EMIMBF_4$**	**2.5**	**23.52**	**4166**	**This work**

Si. no.	Electrode composition	Initial capacity (mAh g^{-1})	Retained capacity (mAh g^{-1})	Current rate (C-rate)	Cycle number	Reference
1	Sulfur - activated carbon composite	800	500	2 C	50 cycles	[8]
2	Sulfur - porous carbon nanoplates composite	1177	762	0.1 C	50 cycles	[9]
3	Sulfur - ketjen black composite	1037	510	0.5 C	500 cycles	[10]
4	Sulfur - GO composite	562	311	0.5 C	600 cycles	[11]
5	Sulfur - ketjen black composite	1204	802	0.2 C	100 cycles	[12]
6	Sulfur - activated carbon foam composite	1000	750	0.2 C	100 cycles	[13]
7	S - BAC composite	53	114	1 C	500 cycles	This work

Table S4. The summary of cyclic stability of Li-S batteries using various reported Li-S cathode materials (sulfur and carbon material composite) with the S-BAC cathode.

References:

1 Z. Liu, H. Zhang, Q. Yang and Y. Chen, *Electrochim. Acta*.

2 Y. Chen, X. Zhang, D. Zhang and Y. Ma, *Mater. Lett.*, 2012, **68**, 475–477.

3 N. das M. Pereira, J. P. C. Trigueiro, I. de F. Monteiro, L. A. Montoro and G. G. Silva, *Electrochim. Acta*, 2018, **259**, 783–792.

4 Z. Lei, Z. Liu, H. Wang, X. Sun, L. Lu and X. S. Zhao, *J. Mater. Chem. A*, 2013, **1**, 2313–2321.

5 W. Lu, K. Henry, C. Turchi and J. Pellegrino, *J. Electrochem. Soc.*, 2008, **155**, A361–A367.

6 A. Paravannoor, S. V Nair, P. Pattathil, M. Manca and A. Balakrishnan, *Chem. Commun.*, 2015, **51**, 6092–6095.

7 A. Balducci, R. Dugas, P.-L. Taberna, P. Simon, D. Plee, M. Mastragostino and S. Passerini, *J. Power Sources*, 2007, **165**, 922–927.

8 H. S. Ryu, J. W. Park, J. Park, J.-P. Ahn, K.-W. Kim, J.-H. Ahn, T.-H. Nam, G. Wang and H.-J. Ahn, *J. Mater. Chem. A*, 2013, **1**, 1573–1578.

9 G. Xu, B. Ding, L. Shen, P. Nie, J. Han and X. Zhang, *J. Mater. Chem. A*, 2013, **1**, 4490–4496.

10 H. Zhou, Q. Tang, Q. Xu, Y. Zhang, C. Huang, Y. Xu, A. Hu and X. Chen, *RSC Adv.*, 2020, **10**, 18115–18123.

11 S. Song, L. Shi, S. Lu, Y. Pang, Y. Wang, M. Zhu, D. Ding and S. Ding, *J. Memb. Sci.*, 2018, **563**, 277–283.

12 J. H. Ahn, H.-J. Shin, S. Abbas, K.-Y. Lee and H. Y. Ha, *J. Mater. Chem. A*, 2019, **7**, 3772–3782.

13 J. Zhang, J. Xiang, Z. Dong, Y. Liu, Y. Wu, C. Xu and G. Du, *Electrochim. Acta*, 2014, **116**, 146–151.
